# A Technique for Fabrication of an Orbital Prosthesis: A Case Report

**DOI:** 10.5681/joddd.2010.018

**Published:** 2010-06-24

**Authors:** Ali Hafezeqoran, Rodabeh Koodaryan

**Affiliations:** ^1^ Assistant Professor, Department of Prosthodontics, Faculty of Dentistry, Tabriz University of Medical Sciences, Tabriz, Iran

**Keywords:** Artificial, eye, ophthalmia, orbit

## Abstract

Rehabilitation of facial defects is a complex task, requiring an individualized design of the technique for each patient. The disfigurement associated with the loss of an eye may result in significant physical and emotional problems. Various treat-ment modalities are available, one of which is the use of implants. Although implant-supported orbital prosthesis has a su-perior outcome, it may not be advisable in all the patients due to economic factors. The present article describes a simplified technique for the fabrication of a silicone orbital prosthesis by constructing a custom ocular prosthesis to achieve ideal fit and aesthetics. Multidisciplinary management and team approach are essential in providing accurate and effective rehabilitation.

## Introduction

The loss or absence of an eye may result from a congenital defect, irreparable trauma, a painful blind eye, sympathetic ophthalmia, or the need for histologic confirmation of a suspected diagnosis.^1 , 2^ The lost orbital volume resulting from removal of the globe can be replaced by integrated or non-integrated orbital implants.^3 , 4^ The placement of a conformer minimizes the changes in the socket size, maintains the shape of the conjuctival fornices, and prevents scar contractures during tissue healing.^5 , 6^ The replacement of the lost eye, as soon as possible after recovery from an eye enucleation procedure, is necessary to promote physical and psychological healing for the patient and to improve social acceptance. Therefore, immediately after tissue healing is complete, the conformer is replaced by a permanent ocular prosthesis. A multidisciplinary management and team approach are essential in providing accurate and effective rehabilitation and follow-up care for the patient.^7^

This article describes a simplified method for the fabrication of a silicone orbital prosthesis.

## Case Report

A 60-year-old woman was referred to the Department of Prosthodontics at Faculty of Dentistry, Shahid Beheshti University of Medical Sciences ( Figure 1). The patient complained of facial disfigurement due to loss of the left eye. A history of exenteration of orbit, which had been carried out one year before due to eradication of meningioma, was noted.

**Figure:**
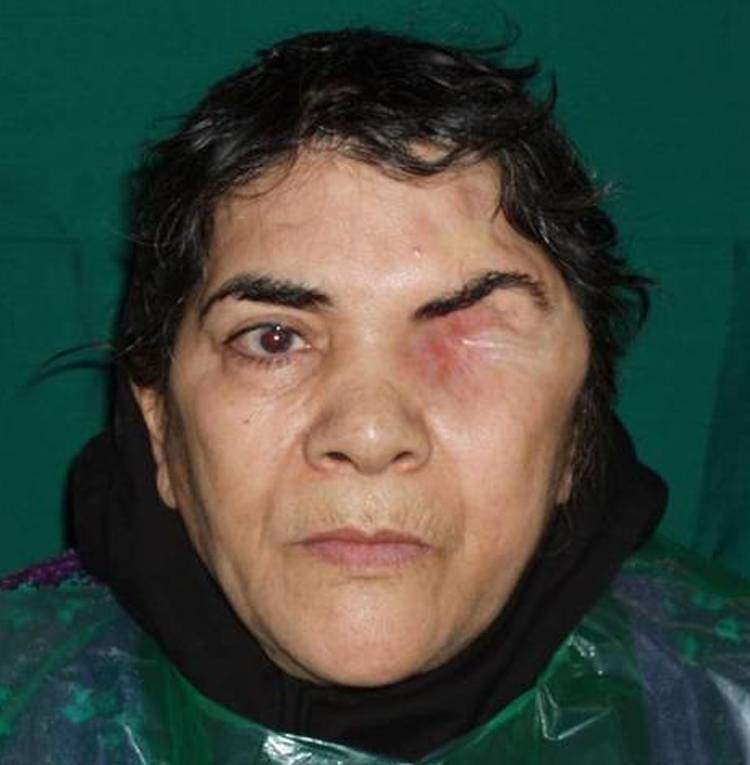
Figure1. Frontal view of the patient before treatment.

### Technique

1) After evaluation and inspection of the anophthalmic socket and defect region, the diameter of the iris and pupil on the intact side was measured using a pair of Boley Gauge callipers.

2) The eyebrow and eyelashes were lightly lubricated. Then an impression was made.

3) A direct impression was prepared according to Mathew’s^8^classification. Irreversible hydrocolloid (Iralgin Alginate, Iran) along with reinforcement by dental plaster (Parsdent, Tehran, Iran) was applied. Subsequently, a cast was poured in dental stone type II (Parsdent, Tehran, Iran) (Figure 2).

Figure 2. A direct impression (a) along with reinforcement by dental plaster (b) was applied.

**a:**
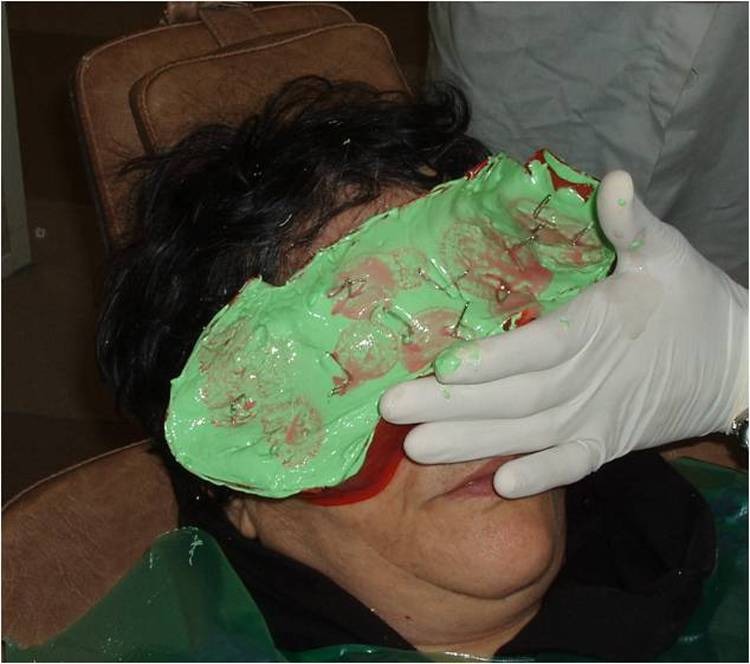

**b:**
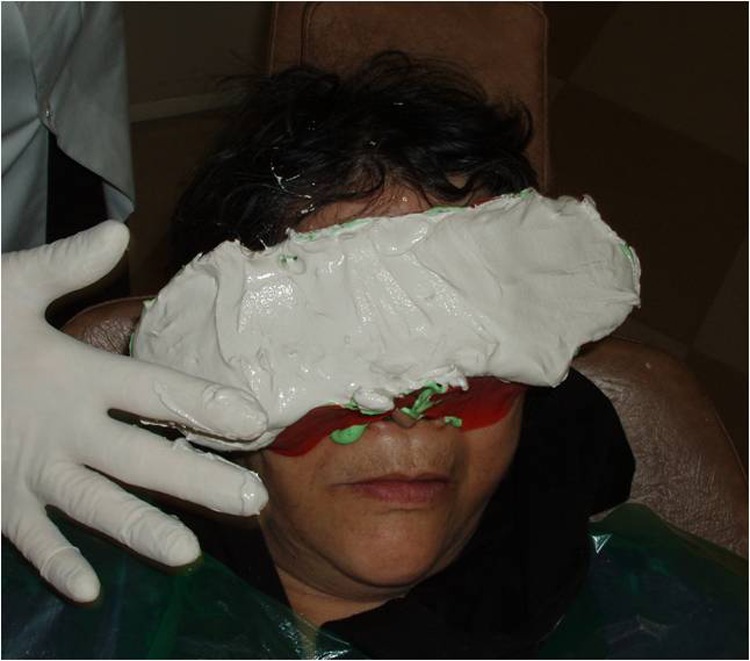

4) A wax model similar to the contour of the globe was constructed for the fabrication of custom ocular prosthesis. This wax model was invested in dental stone type II (Parsdent, Tehran, Iran) and then the mold was packed with acrylic resin mixed with zinc oxide powder (Figure 3).


Figure 3. Wax model similar to the contour of the globe (a) was invested in dental stone (b). A disk with a diameter 1.0 mm smaller than the iris size was prepared (d). A flat surface was prepared in the sclera blank for the iris disk (d, e). Sclera was painted (f).

**a:**
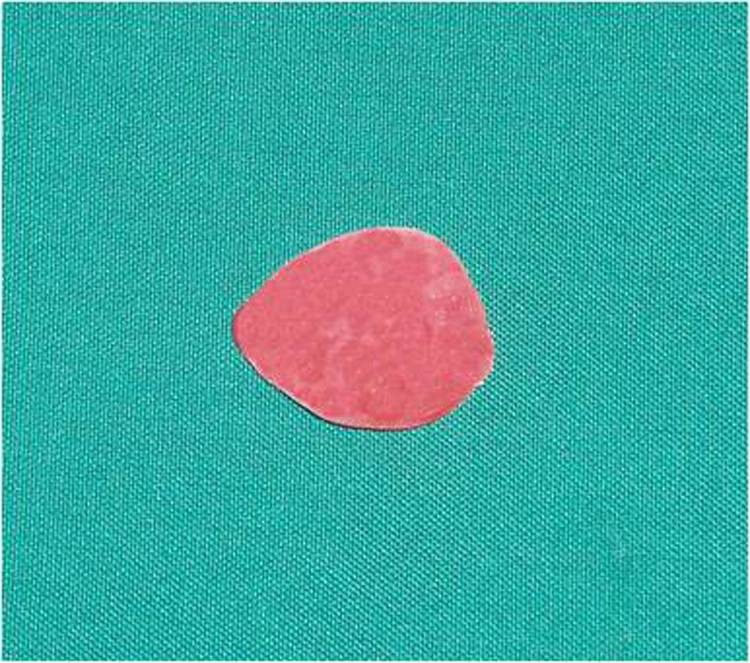

**b:**
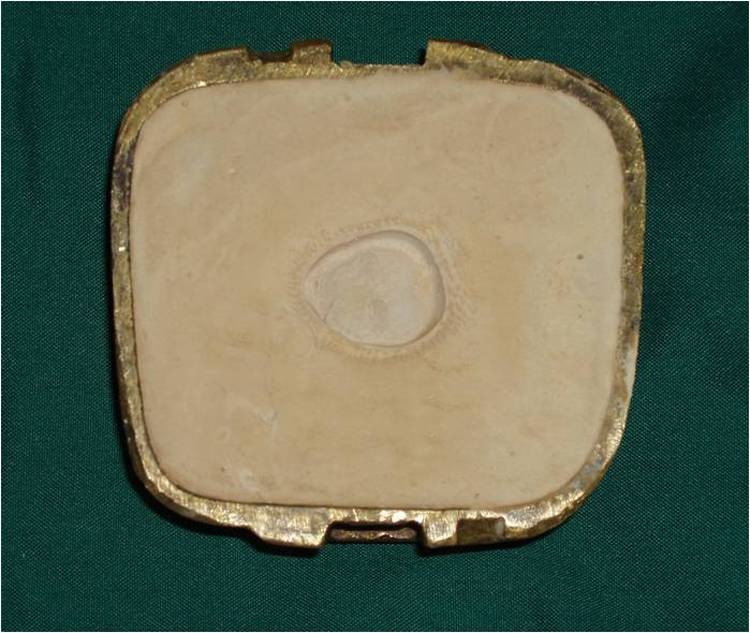

**c:**
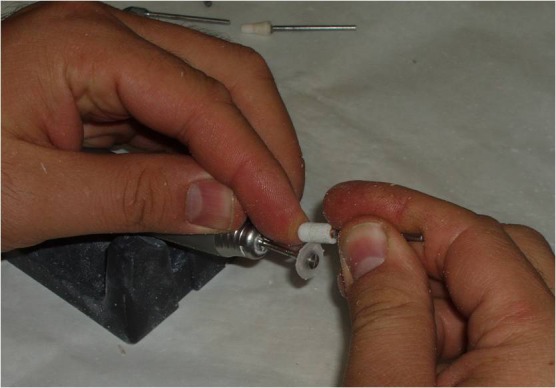

**d:**
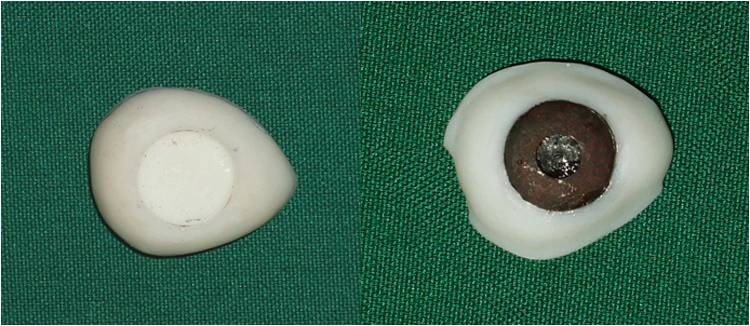

**e:**
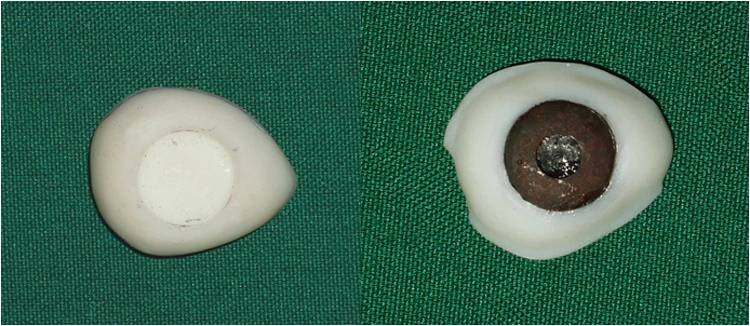

**f:**
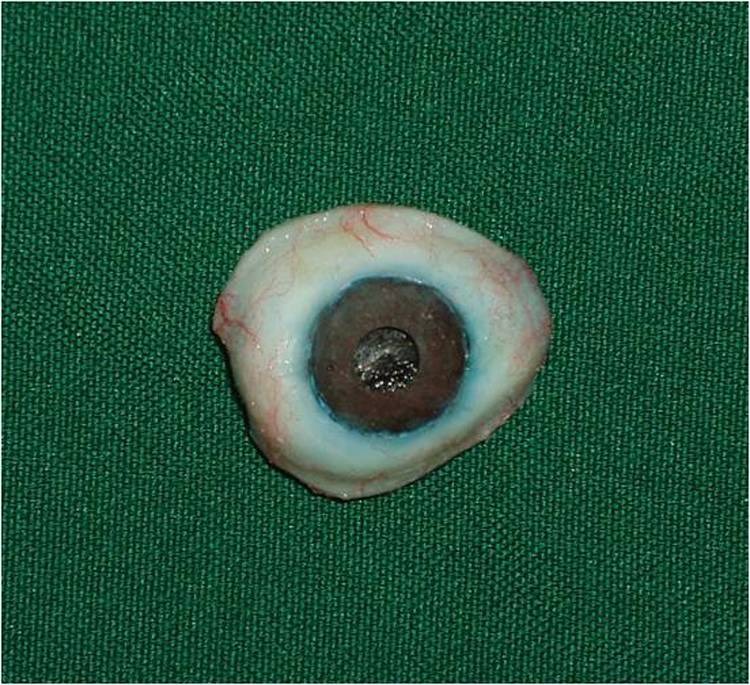

5) An approximately 1.0-mm-thick autopolymerizing acrylic resin (Orthocryl, Dentaurum, Germany) disk with a diameter 1.0 mm smaller than the decided iris size was prepared (Figure 3).

6) Painting the iris disk involves both artistic skills and the science of colour. Many techniques have been described in the literature for painting an artificial iris. The acrylic-based pigments were used to paint the disk. The painted iris disk was checked for colour accuracy against the natural eye by placing a drop of water on the painted surface during construction.

7) Using a flat-end bur, a flat surface was prepared in the sclera blank for the iris disk. An ocular button was luted to the prepared flat surface and the assembly was tried in. Its orientation was adjusted while the patient looked directly into the observer's eye (Figure 3).

8) The flat part of the prosthesis was painted with the base colour of the iris, and a black dot was placed for the pupil.

9) The limbus or the fuzzy demarcation between the iris and sclera was painted. The remainder of the prosthesis was then painted to match the sclera of natural eye. Blood vessels were placed on the sclera using red embroidery floss (Figure 3).

10) A scraper was used to create the corneal prominence in the mold space. Sclera blank was placed in the flask, and clear ocular acrylic resin (Orthocryl, Dentaurum, Germany) was mixed and placed in the mold space. A thin layer of processed clear acrylic resin was applied over the surface of the painted sclera. Then the prosthesis was trimmed and polished (Figure 4).


Figure 4. Clear ocular acrylic resin was placed into the mold space (a). A temporary base of acrylic resin was formed on the working cast (b). The prosthesis was processed (c) and trimmed (d).

**a:**
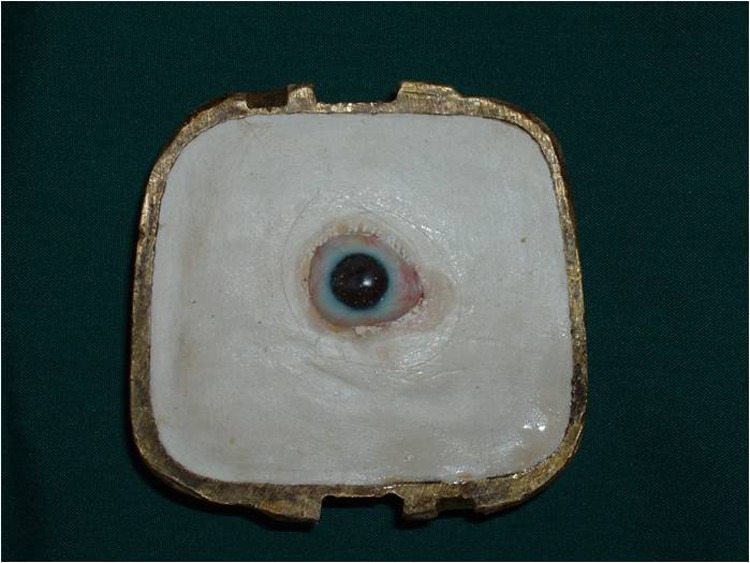

**b:**
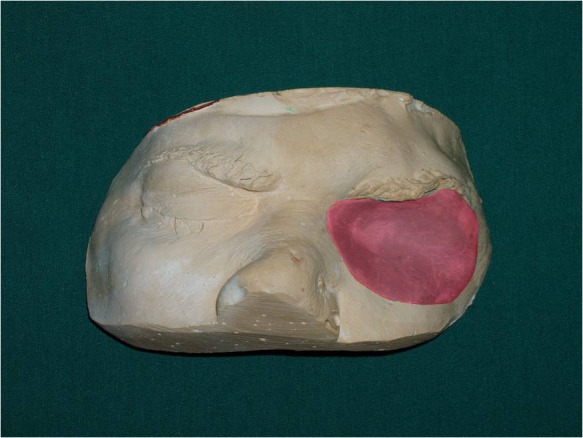

**c:**
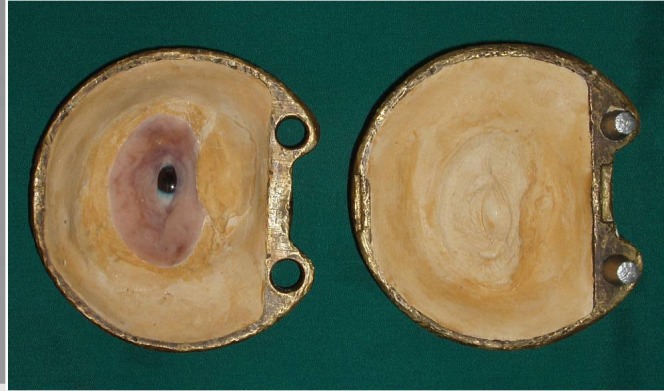

**d:**
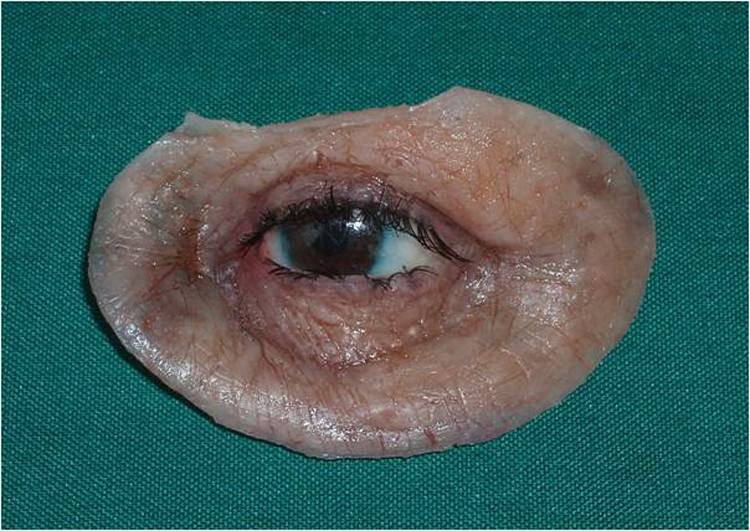

11) A temporary base of acrylic resin was formed on the working cast. This base formed the basis for positioning the ocular section of the prosthesis within the defect in the same planes as the normal eye (Figure 4).

12) The assembly was placed in the orbital defect and manipulated into the position corresponding to the normal eye gaze. The eyelid aperture was established by softening and placing two small strips of wax over the ocular section for primary evaluation (Figure 5).


Figure 5. Frontal view of patient after treatment (a). The assembly was placed into the orbital defect for primary evaluation (b).

**a:**
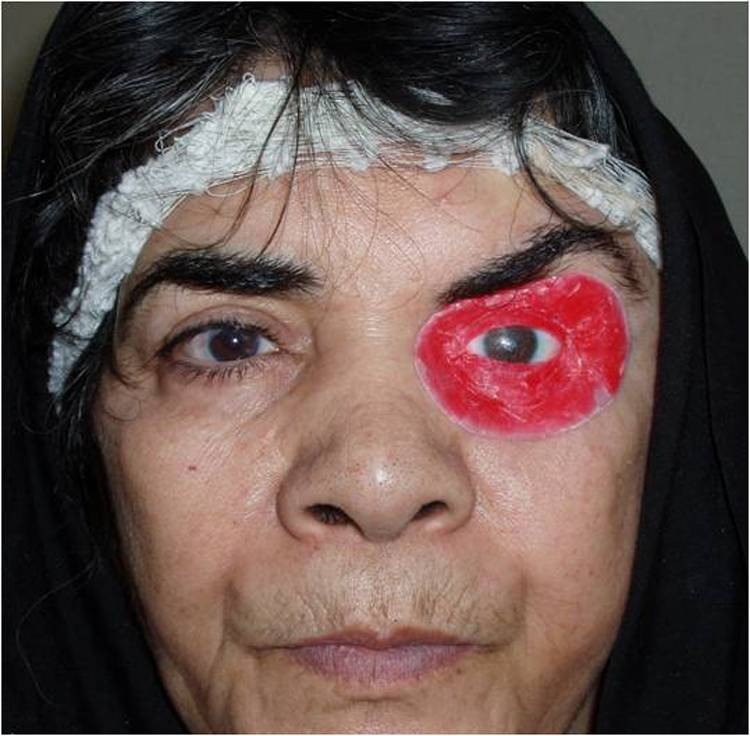

**b:**
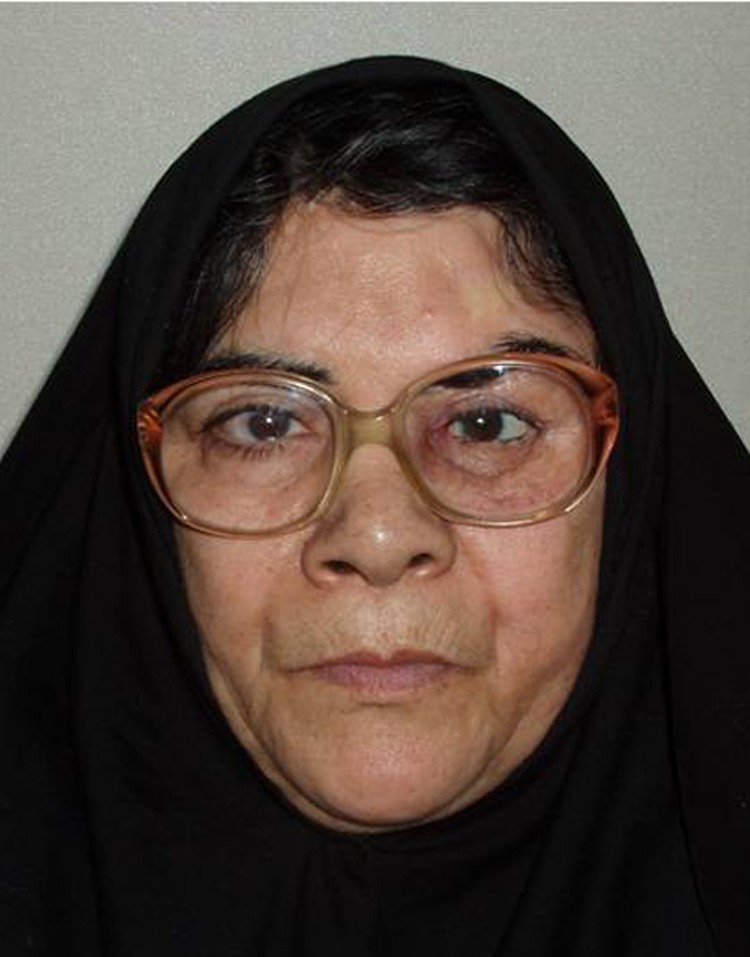

13) After establishment of the correct position of the ocular section, a mix of light-body silicon (Speedex, Asia Chemi Teb Co, Tabriz, Iran) material was applied on the established assembly to stabilize the pupil position.

14) On the back of the sclera portion three notches, with a depth of 1 mm, were prepared to transfer the same orientation back into the mold. After applying a lubricant, autopolymerizing acrylic resin was placed on the base. Therefore, by incorporating these indices the orientation of the ocular segment of the prosthesis could be kept the same and the ocular segment could be separated and then placed at the same orientation.

15) After sculpturing the prosthesis pattern, the final surface contour and skin texture were established by carving in lines and wrinkles found around the normal eye. Pressing a wet piece of gauze square into softened wax will produce a texture similar to normal skin.

16) The wax pattern was flasked; after boil-out, the temporary base was removed and trimmed 1 mm short of prosthesis border. Then a cope of a second mold was placed in position and the second pour of mold was made over the shortened base. After the stone material was set, the definitive base was processed with heat-cured acrylic resin (Meliodent Multicryl, Heraeus-kulzer GmbH, Wehrheim, Germany).

17) Using intrinsic coloration, the prosthesis was processed with the use of the first mold assembly. After the polymerization was complete, the residual flush was trimmed back with a scalpel and finished with an abrasive stone (Figure 4).

18) The patient was instructed in the use and care of the prosthesis (Figure 5).

## Discussion

Lost eyes have been replaced with prostheses for many years in the form of stock or custom ocular prosthesis. Often, however, a custom-made ocular prosthesis which provides a more precise and satisfactory esthetic appearance is indicated, especially for those who have lost ocular structures through orbital evisceration or orbital enucleation.^1^ In the treatment of a patient requiring a custom ocular prosthesis many successful techniques are available to the practitioner. Although implant-retained ocular prostheses play an important role in the success of treatment, conventionally retained orbital prostheses are practical, trouble-free, cost-effective, and successful.^3^
